# To Be or Not To Be Translational

**Published:** 2013-01-04

**Authors:** A Anastasio, A Armenante, A Gimigliano, A Moscarino, S Panzera, R Romano, I Russo, G Scarpati

**Affiliations:** Master Course RISS, University of Salerno

**Keywords:** Translational medicine, Translational research

## Abstract

Translational Research means different things to different people, but it seems crucial to almost everyone. This discipline, although defined differently in academia, regulatory institutions, and industry, shares the fundamental vision of Translational Medicine, which efficiently and effectively translates basic scientific findings relevant to human disease into knowledge that benefits patients. In the present perspective, we collected commentaries and descriptions about Translational Medicine to stimulate discussion and better understand what Translational Medicine is.

There is growing evidence of the importance of translational science and medicine in the improvement of patient outcome, even though the definitions of translational medicine need to be further clarified.

Although a Medline search indicates that the term “translational research” appeared as early as 1993, there were relatively few references to this term in the medical literature during the 1990s, and most references were to research about cancer. Today, the literature includes a plethora of attempts in various fields to define the term. Translational medicine is an emerging area comprising multidisciplinary research from basic sciences to medical applications well summarized by the Bench-to-Beside concept; this entails close collaboration between clinicians and basic scientists across institutes. Subsequently Marincola clarified that Translational Medicine should be regarded as a two-way road: Bench-to-Bedside and Bedside-to-Bench [1].

Recently Wang et al. wrote: “Clinical and translational medicine are expected to include scientific and regulatory investigations to translate preclinical researches to clinical application with a specific emphasis on new biotechnologies, biomaterials, bioengineering, disease-specific biomarkers, cellular and molecular medicine, bioinformatics, applied immunology, molecular imaging, drug discovery and development, and regulation and health policy. It is believed that clinical and translational medicine will benefit and improve novel diagnostics/prognostics and therapeutics for clinical use, post-genomic knowledge and experience, and/or new disciplines that reflect additional levels of complexity” [2].

Over the past 30 or so years, the ecosystems of basic and clinical research have diverged. An interesting article of D. Butler reports: “The abyss left behind is sometimes labelled the ‘valley of death’ — and neither basic researchers, busy with discoveries, nor physicians, busy with patients, are keen to venture there” [3].

Finally, “The clinical and basic scientists do not really communicate,” says Barbara Alving, director of the NIH’s National Center for Research Resources in Bethesda [3].

In the present perspective, we collected commentaries and descriptions about clinical and translational medicine from some members of Master RISS (a master’s degree in translational medicine of the University of Salerno, Italy) to stimulate discussion and improve the understanding.

*Antonio Anastasio* (MSc, medical biotechnology): When I was asked to write a definition of translational medicine I smiled because I don’t believe to be an expert to know how answering to this question. I think that translational medicine isn’t an autonomous discipline, but one of the ways to do a good search, a search with a capital S. I simply believe that a good research is passion, sacrifice, hope. The research is waiting a result that fails or that makes you exult as a child. Research is the thing that makes you wake up every morning and, once arrived in the laboratory, makes you have butterflies in your stomach. The search is simply my life.

*Annunziata Armenante* (PhD, industrial biotechnology): I am a standard researcher of the bench so for me the translational medicine is the capability to use knowledges, discoveries and learning of basic and clinical research to solve problems of medicine or to improve diseases diagnoses. A historical example is the accidental discovery of *Penicillium* by Alexander Fleming. Today it’s difficult to make startling discoveries because enormous progresses are been made by human society, but there is a lot to discover yet. Therefore, the translational medicine is the effort that the medicine must make to leave the old patterns. Today vocational training is more and more sectorial, only multi-disciplinary approaches and the ability to transfer information and discoveries from biology and technology to medicine are the step which will allow the true progress of society.

*Anna Gimigliano* (PhD, medical biotechnology): Translational medicine represents a medical research model bridging basic research with clinical application of experimental findings to patient’s diagnosis and therapy. This model assumes researchers can strictly collaborate both on conceptual and methodological aspects of multiple disciplines to develop a network of integrated knowledge. An innovative translational framework has to stimulate academic institutions, clinical departments and private companies to share scientific projects and to fund chances in order to elucidate the mechanisms involved in human diseases. Such approach can enhance the quality of biomedical research leading to a more efficient development of new biomarkers and drugs. Therefore, the optimization process of human and financial resources will improve the competitiveness of public health system towards the challenges of personalized medicine.

*Azzurra Moscarino* (MSc, pharmaceutical chemistry and technology): Translational medicine is a way to overcome the extreme specialization that we have built over the years, that broke the way to see the research and prevented us from reaching a definitive solution, offering only partial solutions of pathology. Translational medicine is for me like a triathlon competition, where all athletes despite their diversity are competing for the same purpose.

*Stefania Panzera* (MSc, pharmaceutical chemistry and technology): The concept of translational medicine in my opinion implies evolution in thinking and organizing research centers, especially university students, in order to create a continuous flow of knowledge and bidirectional operation that goes from the laboratory to the bedside. Root is the collaboration between various professionals, each skilled and specialized in their field, knowledge of which can certainly be transformed into an added value as well as economic ensuring concrete results in even shorter time.

*Rosalba Romano* (MD, anesthesia): The definition of “translational medicine” is still in progress. It is closely connected to the necessity to share knowledge among researchers, physicians and public. The act of translating is primary in filling the gap between basic science and clinical research, and between clinical research and the daily clinic. The spread of the principle of communication among different professional groups is the only way to make it possible. Basic science research can’t be aimless, but need to be oriented to specific problems. At the same time, the improvement of the research is not enough because the translating process must involve the public and the last users of knowledge. The translational medicine is a new approach to health, an organized point of view of research from “bench to bedside” and of its understanding using communication among researchers of different cultural backgrounds and representatives of medical and non- medical disciplines.

*Ilaria Russo* (PhD, medical biotechnology): Translational is looking to man as an infinite set of molecules, receptors, that interact in a finely organized system. This imply, that the biologist or any researcher that studies different human pathologies (biologists, chemists, mathematicians, physicists), know the mechanisms that underlie the physiology of the human body and define the different issues in one unique interpretation. In this context, there must be a continuous dialogue between different researchers to transfer the new skills and knowledge. Along this line, the translational medicine is before training and then it’s action and project implementation to identify the new therapeutical strategies for the human health. This represent the real strategy for an effective resolution of the most urgent medical problems that affect the society promoting the scientific progress and technology using also the political and economic strategies. In this vision of medicine, the man represents “the origin and the end” of all scientific knowledge.

*Giuliana Scarpati* (MD, anesthesia): Translational Medicine is an inter-disciplinary science. Modern research emphasizes the development of targeted and personalized therapies aimed at treating patients according to our modern understanding. In this context, translational medicine is not a magic world which includes all sciences, but a tool to enhance the efficiency of research integrating areas of expertise through a broad spectrum of disciplines.

In conclusion, it is necessary and critical to understand the definition and concept of clinical and translational medicine and allow the variations of understanding, but it is equally clear from the stated above that there is no single definition of translational medicine.
